# Genome-Wide Microarrray Analysis Reveals Roles for the REF-1 Family Member HLH-29 in Ferritin Synthesis and Peroxide Stress Response

**DOI:** 10.1371/journal.pone.0059719

**Published:** 2013-03-22

**Authors:** Thanh K. Quach, Han Ting Chou, Kun Wang, Gaolin Zheng Milledge, Casonya M. Johnson

**Affiliations:** 1 Department of Biology, College of Arts and Sciences, Georgia State University, Atlanta, Georgia, United States of America; 2 Department of Environmental Health Science, Division of Biostatistics, New York University School of Medicine, New York, New York, United States of America; 3 Department of Mathematics and Computer Science, North Carolina Central University Durham, North Carolina, United States of America; University of South Florida, United States of America

## Abstract

In *Caenorhabditis elegans*, the six proteins that make up the REF-1 family have been identified as functional homologs of the Hairy/Enhancer of Split (HES) proteins. These transcription factors act in both Notch dependent and Notch-independent pathways to regulate embryonic events during development; however, their post-embryonic functions are not well defined. As a first step toward understanding how the REF-1 family works together to coordinate post-embryonic events, we used gene expression microarray analysis to identify transcriptional targets of HLH-29 in L4/young adult stage animals. Here we show that HLH-29 targets are genes needed for the regulation of growth and lifespan, including genes required for oxidative stress response and fatty acid metabolism, and the ferritin genes, *ftn-1* and *ftn-2*. We show that HLH-29 regulates *ftn-1* expression via promoter sequences upstream of the iron-dependent element that is recognized by the hypoxia inducible factor, HIF-1. Additionally, *hlh-29* mutants are more resistant to peroxide stress than wild-type animals and *ftn-1*(RNAi) animals, even in the presence of excess iron. Finally we show that HLH-29 acts parallel to DAF-16 but upstream of the microphthalmia transcription factor ortholog, HLH-30, to regulate *ftn-1* expression under normal growth conditions.

## Introduction

The Hairy/Enhancer of Split (HES) transcription factors, members of the basic Helix-Loop-Helix (bHLH) superfamily, coordinate cellular and environmental signals to direct cellular proliferation and tissue morphogenesis. Through physical interactions with multiple classes of transcription factors and transcriptional co-repressors, HES proteins regulate a large number and variety of target genes [1], [2], [3], [4] that are critical for both embryonic development and homeostasis in the adult. In mammals, mutations in HES genes result in muscular, neural, and cardiovascular defects, and have been associated with tumors of the breast, colon, eye, and lung [5], [6], [7], [8], [9]. Despite the prominent role of HES proteins in human disease and in plant and animal development, little is known about the transcriptional networks that they control or about how much overlap there is between the networks of different HES proteins.

In *Caenorhabditis elegans*, the six proteins that make up the REF-1 family have been identified as functional homologs of the HES proteins. One of the two bHLH domains that are characteristic of the REF-1 family proteins shows significant sequence similarity to the bHLH domain of HES proteins. Like the HES proteins, members of the REF-1 family are all involved in embryonic Notch/ligand interactions [10], [11], [12], they all appear to regulate specification and development of the mesoderm and endoderm [13], [14], and they act in both Notch-dependent and Notch-independent functions [15], [16], [17]. Though the REF-1 family lacks the Orange domain that is common to HES proteins in all other organisms, it is possible that the second bHLH domain found in members of the REF-1 family functionally substitutes for the Orange domain to facilitate and stabilize protein-protein interactions.

Most of the available functional data on the REF-1 family involves REF-1, the first member to be identified in genetic screens [18]. REF-1 affects the V ray lineage in *C. elegans* males [13], controls the specification and generation of embryonically generated neurons [19], and is required for the Notch induced left/right asymmetry of the developing embryonic intestine [17], [20]. Molecular studies of the REF-1 family genes *hlh-29* and *hlh-28* indicate that their gene products are identical, and that loss of *hlh-29/hlh-28* activities affects *C. elegans* embryonic viability, egg-laying, and chemorepulsive behaviors [21]. Ectopic expression of *hlh-29* has also been shown to partially rescue the intestinal asymmetry defects associated with mutations in *ref-1* [17]. More recently, HLH-29 has been found to act within the IP_3_ signaling pathway to regulate the ability of oocytes and fertilized eggs to enter and exit the spermatheca, respectively [22], a finding which underscores the importance of the REF-1 family proteins in regulating both embryonic and post-embryonic events. Candidate target genes for HLH-29 have been identified based on the individual DNA binding site selectivity of homodimeric protein [48]; functional annotation of those genes suggests HES-like roles for HLH-29 in regulating embryonic and larval development, growth, cell fate specification, and reproductive behaviors.

As a first step in understanding how the REF-1 family coordinates developmental and reproductive events, in particular those events that occur post-embryonically, we performed genome-wide gene expression analysis using young adult *hlh-29* mutants. Our data suggest that in young adult animals, HLH-29 affects significantly fewer biological processes than previously predicted, and that, for genes that are directly targeted, HLH-29 may function primarily as a heterodimer or via a mechanism that does not involve binding with DNA. Many of the genes in the HLH-29 regulatory network act downstream of insulin/IGF-1 signaling (IIS) to influence aging, the oxidative stress response, and energy metabolism, including the *C. elegans* ferritin genes, *ftn-1* and *ftn-2*.

Ferritins are proteins that act to maintain iron homeostasis by sequestering and storing iron until free iron levels are lower than optimal [23], [24], [25], [26], [27]. The role of ferritins is particularly important in light of the wide range of effects iron has on cellular growth and proliferation, and on human health. Excess iron can catalyze the formation of reactive oxygen species (ROS) [24], and as a result, increase the occurrence of DNA and protein damage [28], while iron deficiency causes cell cycle arrest [29], [30]. Thus, ferritins are critical molecules that facilitate rapid cellular responses to dynamic changes in the levels of environmental iron. Iron homeostasis, then, is maintained in part by regulating the level of ferritin, and this regulation occurs in response to iron and oxygen at the transcriptional and post-transcriptional levels [31], [32], [33].

The *C. elegans* ferritin genes are transcriptionally regulated in response to iron via a cis-regulatory, iron-dependent element (IDE) [34], [35]. Though both genes code for proteins with strong similarity to the mammalian heavy ferritin subunit [23], only one protein, FTN-1, is thought to play a prominent role in iron homeostasis and detoxification [34], [36]. Repression of *ftn-1* transcription in an iron-dependent manner is mediated by the IDE-binding hypoxia-inducible factor, HIF-1 [37], a protein whose mammalian homologs mediate intestinal iron transport during iron deficiency [37]. Intestinal expression of *ftn-1* is mediated by the GATA-type transcription factor, ELT-2, which also binds to sequences within the IDE [37]. Transcriptional regulation of *ftn-1* also involves insulin/IGF-1 signaling, via the FoxO transcription factor DAF-16 [38] and the basic helix loop helix (bHLH) protein, MDL-1 [38]; however these factors do not mediate iron-responsiveness of *ftn-1*. Here we identify HLH-29 as a negative regulator of *ftn-1* transcription via a mechanism that is distinct from the iron-responsive, HIF mediated pathway.

## Results

### Genes in the HLH-29 Regulatory Network are Required for Lifespan, Growth, and Stress Response

Our previous work indicated that HLH-29 is expressed throughout the *C. elegans* lifespan, and that aberrant *hlh-29* expression affects hermaphrodite development and reproduction [21], [22]. We wished to identify the post-embryonic targets of *hlh-29*, and so we used late larval stage (L4)/young-adult animals that were fertile and producing gametes, but were not yet producing fertilized embryos. When gene expression in *hlh-29* mutants was compared to expression in age-matched wild-type animals, we found that the expression of 284 genes was altered by at least 2.0 fold in *hlh-29* mutants. Of these, 250 genes were up-regulated and 34 genes were down-regulated (Table S1). HLH-29 dependent expression for five of the genes was previously reported [22], and we used RT-qPCR to confirm that expression of 21 additional genes was significantly affected in *hlh-29* mutants (Table 1). We found that expression of all of the tested genes was significantly affected in *hlh-29* mutants, and that the direction of regulation (up-regulated versus down-regulated) was the same for 20 out of the 26 tested genes. Interestingly, only seven of the targets identified in our microarray correlated with previously predicted targets of homodimeric HLH-29 [48]. These genes were *ftn-1*, *gst-10*, *mtl-1*, *sodh-1*, F07A5.2, F53H1.1, and F59A2.5. Hypergeometric analysis shows that the number of *hlh-29* targets from this study is somewhat under-represented in the previously predicted list of *hlh-29* targets when compared to a randomly chosen gene set of the same size (P-value = 0.0997). Three possibilities may explain why there is not a larger correlation between the two sets of genes. First, many of the genes whose promoters contain the predicted HLH-29 recognition sequences may be regulated by HLH-29 at developmental stages not used in this study. Second, some of the genes identified in this study may be direct targets of HLH-29 but regulated via a mechanism that does not require the predicted recognition sequence. For example, HLH-29 may act at some targets as a heterodimer that recognizes a different binding sequence from the one predicted for the homodimeric protein. Alternatively, HLH-29 may act at those promoters without actually making contact with the DNA, perhaps in a complex with other proteins. Third and finally, many of the genes identified in this study are likely to be indirectly regulated by HLH-29, and so would not be expected to contain recognition sequences for HLH-29 within their promoters.

**Table 1:** Validation of HLH-29 Targets by RT-qPCR.

WormBase ID	Sequence ID	Gene Name	log2 Fold Change (microarray)	log2 Fold Change (RT-qPCR)
WBGene00009221	F28F8.2	*acs-2*	2.69821	1.94485
WBGene00000230	F27C1.7	*atp-3*	1.01436	0.83592
WBGene00000169	F32A5.5	*aqp-1*	2.09085	0.59741
WBGene00016509	c37h5.6	C37H5.6**	1.30451	0.56150
WBGene00016052	C24B9.9	*dod-3*	1.50589	0.27917
WBGene00010305	F59A2.5	F59A2.5Δ	−1.39506	−1.64837
WBGene00001394	W02A2.1	*fat-2*	1.83592	0.25701
WBGene00001500	C54F6.14	*ftn-1* Δ	2.87381	1.50538
WBGene00001564	C05E4.9	*gei-7*	1.47249	0.40163
WBGene00001685	K10B3.7	*gpd-3*	1.17664	1.82862
WBGene00001758	Y45G12C.2	*gst-10* Δ	−1.32884	−0.41922
WBGene00020930	W02C12.3	*hlh-30*	1.17664	0.78586
WBGene00003242	C37C3.6	*mig-6* **	1.44890	2.79840
WBGene00003421	F09E8.3	*msh-5*	−1.20477	−0.64837
WBGene00003473	K11G9.6	*mtl-1* Δ	1.83754	0.63087
WBGene00008149	C47E12.4	*pyp-1* **	1.35614	1.53306
WBGene00004736	K11D9.2	*sca-1* **	1.40054	0.86235
WBGene00010790	K12G11.3	*sodh-1* Δ	1.71896	0.72683
WBGene00011733	T12D8.5	T12D8.5	1.94486	0.84960
WBGene00014095	ZK829.4	*gdh-1*	1.33171	1.08338
WBGene00007601	C15C6.2	C15C6.2	1.40054	−0.31294
WBGene00015102	B0280.5	*cpg-2*	1.08159	−1.35719
WBGene00015044	B0213.15	*cyp-34A9*	1.18587	−0.26882
WBGene00001263	K04H4.1	*emb-9* **	1.22651	−0.87567
WBGene00003022	ZK418.4	*lin-37*	1.09356	−1.20091
WBGene00004078	F52E1.1	*pos-1*	1.24791	−2.60943

** RT-qPCR results for these genes were previously published [22].

Δ These genes correlate with previously predicted targets of homodimeric HLH-29 [48].

**Figure 1:**
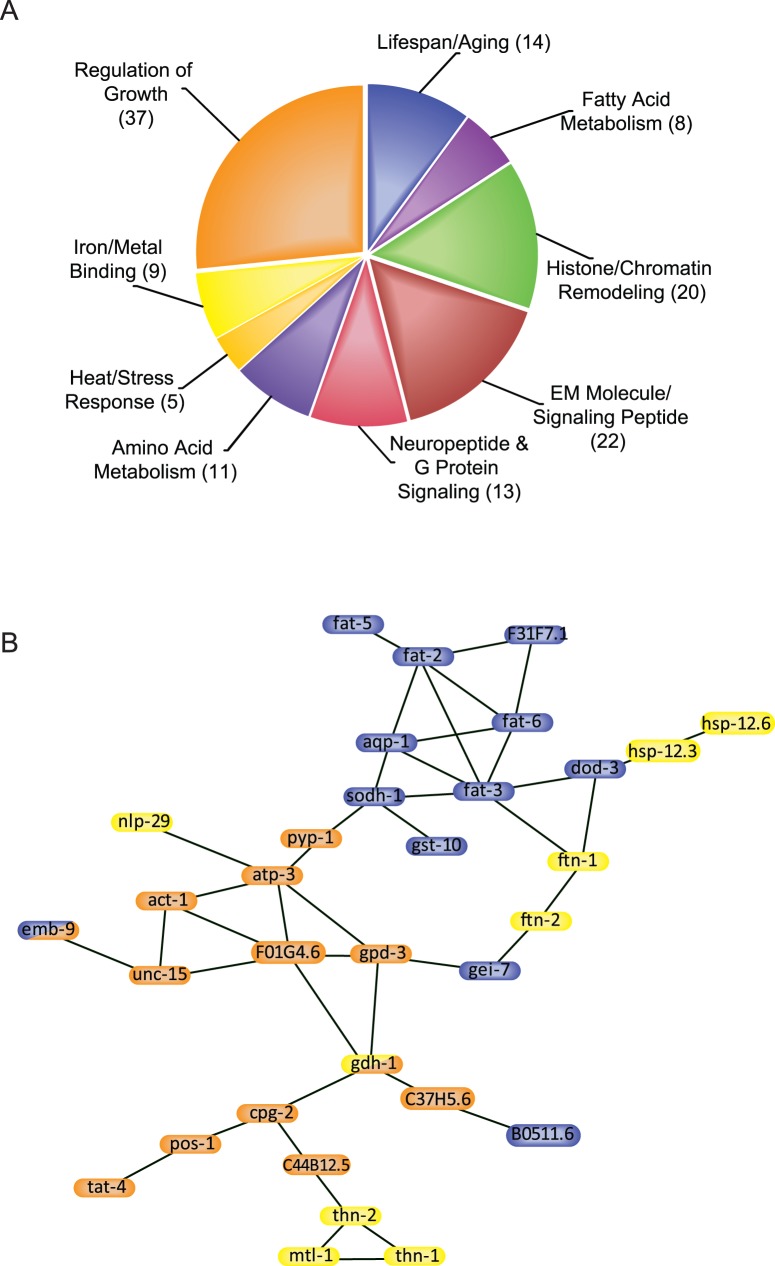
HLH-29 Regulatory Network. A) Functional annotation and distribution of HLH-29 targets as predicted by DAVID analysis. Brackets indicate the number of target genes in each cluster. B) Protein interaction network of HLH-29 targets that associate with the GO terms “lifespan and aging” (blue ovals), “stress response” (yellow ovals), or “growth” (orange ovals), as predicted by STRING 9.0.

We used the Database for Annotation, Visualization and Integrated Discovery (DAVID), version 6.7, to cluster related target genes based on enriched Gene Ontology (GO) terms [39], [40]. As shown in Table S2, 256 of the 284 HLH-29 target genes were assigned to 30 functional clusters; 136 genes were grouped together in the first ten clusters (Figure 1A). Approximately 1/3 of the genes affected by loss of hlh*-29* activity (75 of 256) were associated with at least one of the following GO terms: ‘Response To Metals/Toxins’, ‘Response To Stress’, ‘Lifespan and Aging’, or ‘Growth’. We used the Search Tool for the Retrieval of Interacting Genes/Proteins (STRING 9.0) [41] to identify predicted interactions among those 75 gene products. Thirty-three of the 75 were in a single interaction module (Figure 1B) with few links between nodes. FAT-2, FAT-3 and ATP-3, each with more than four links to other nodes, were considered the only hubs in this module. Based on the GO analysis, FAT-3 bridges those proteins affecting ‘Lifespan and Aging’, while ATP-3 bridges those controlling ‘Growth’. Proteins needed for ‘Response to Stress’ and ‘Response to Metals/Toxins’ were distributed between the two hubs.

### HLH-29 Target Genes are Downstream of DAF-16

As shown in Table S3, thirty-two genes known to be regulated by DAF-16 [42] were affected by loss of *hlh-29* activity. By hypergeometric analysis, this number of *hlh-29* targets is significantly over-represented among DAF-16 targets when compared to a randomly chosen gene set of the same size (P-value = 8.51×10^−12^). DAF-16 is a FOXO transcription factor that, in addition to regulating *ftn-1* expression [38], acts in the insulin/IGF-1-mediated signaling (IIS) pathway to regulate dauer formation, longevity, fat metabolism, stress response, and innate immunity [43], [44], [45], [46]. DAF-16 target genes are separated into two classes. Class I genes are up-regulated in *daf-2* mutants and down-regulated in *daf-2*;*daf-16* double mutants. Class II genes are down-regulated in *daf-2* mutants, and up-regulated in *daf-2;daf-16* double mutants [42]. We found that the expression of 28 Class I genes was up-regulated in *hlh-29* mutants, while the expression of four Class II genes was down-regulated. This result suggests that HLH-29 antagonizes DAF-16 signaling in the cell, though expression of neither *daf-16* nor *daf-2*, the insulin receptor gene, was affected in *hlh-29* mutants (Figure S1A). Many of the target genes shared by DAF-16 and HLH-29 are required for normal responses to oxidative stress. We found 12 additional, DAF-16 independent, *hlh-29* target genes that are regulated in response to oxidative stress and aging [47]. These data suggest that HLH-29 functions either downstream of or parallel to DAF-16 to regulate genes required for stress response. The presence of predicted HLH-29 binding sites in the promoters of the DAF-16 target genes, *ftn-1*, *mtl-1*, and *sodh-1* [48] supports the possibility that HLH-29 is antagonizing DAF-16 activity at their shared targets through a separate pathway rather than through a single pathway.

### HLH-29 Acts Upstream of HLH-30 and Genes Required for Energy Homeostasis

Though our microarray results show that a number of HLH-29 targets are required for nucleosome stability and chromatin remodeling, the only known transcription factor gene to be affected in *hlh-29* mutants was *hlh-30*. HLH-30, an ortholog of the mammalian microphthalmia transcription factor family protein, TFE3 [49], regulates genes involved in metabolism and fat storage [48]. Expression of *hlh-29* and *hlh-30* overlap in adult animals in the intestines, spermatheca, and vulva muscles [21], [48], and *hlh-29* mutants were previously shown to increase fat storage [21]. The activity of *hlh-30* increases in *hlh-29* mutants, and 20 genes, five of which are required for fatty acid metabolism or biosynthesis, were inversely affected in *hlh-29* mutants and *hlh-30* mutants (Table S4). The *hlh-29* targets are significantly over-represented in *hlh-30* targets when compared to a randomly chosen gene set of the same size (p-value = 3.81123×10^−13^). Though *hlh-29* was not identified as a target of *hlh-30* in a previous study [48], we found that *hlh-29* mRNA levels increase in *hlh-30* (RNAi) animals (Figure S1B).

We also found that HLH-29 affects the expression of HLH-30 independent genes that are required for fatty acid synthesis and oxidation. Expression of two of the three *C. elegans* fatty acid Δ9-desaturase genes, *fat-5* and *fat-6*, the Δ12 desaturase gene, *fat-2*, and the Δ6 desaturase gene, *fat-3*, is up-regulated in *hlh-29* animals. Additionally, the acyl-CoA synthetase gene, *acs-2*, the glutamine synthetase gene, *gln-3*, and the aquaporin glycerol transporter gene, *aqp-1*, are affected. This finding correlates well with the expression of HLH-29 in the intestines [21], the major site of fat storage in *C. elegans* [50] and the fat storage phenotype of *hlh-29* mutants, and further underscores a role for HLH-29 in mediating energy homeostasis.

### HLH-29 Influences *ftn-1* Levels but not Iron-Responsiveness

A number of genes in the HLH-29 regulatory network are required in *C. elegans* for heme uptake and utilization [51], which raises the possibility that HLH-29 plays a role in maintaining iron homeostasis (reviewed in [52], [53]). In addition to *ftn-1* and *ftn-2*, these genes include the cytochrome P450 genes, *cyp-13A5*, *cyp-13A6*, *cyp-13B1*, and *cyp-34A9* [54]; the glutathione-S-transferase genes, *gst-10*, *gst-15*, and *gst-19* [55]; and the heme permease gene, *hrg-1* [56]. Because a number of genes affected by loss of *hlh-29* activity are regulated by oxidative stress and aging (Table S3B) or have oxido-reductase activities (Table S3C), and because ferritins function, in part, to minimize oxidative stress by sequestering and oxidizing free cellular iron [57], [58], we further characterized the role of HLH-29 in regulating *ftn-1* expression.

**Figure 2:**
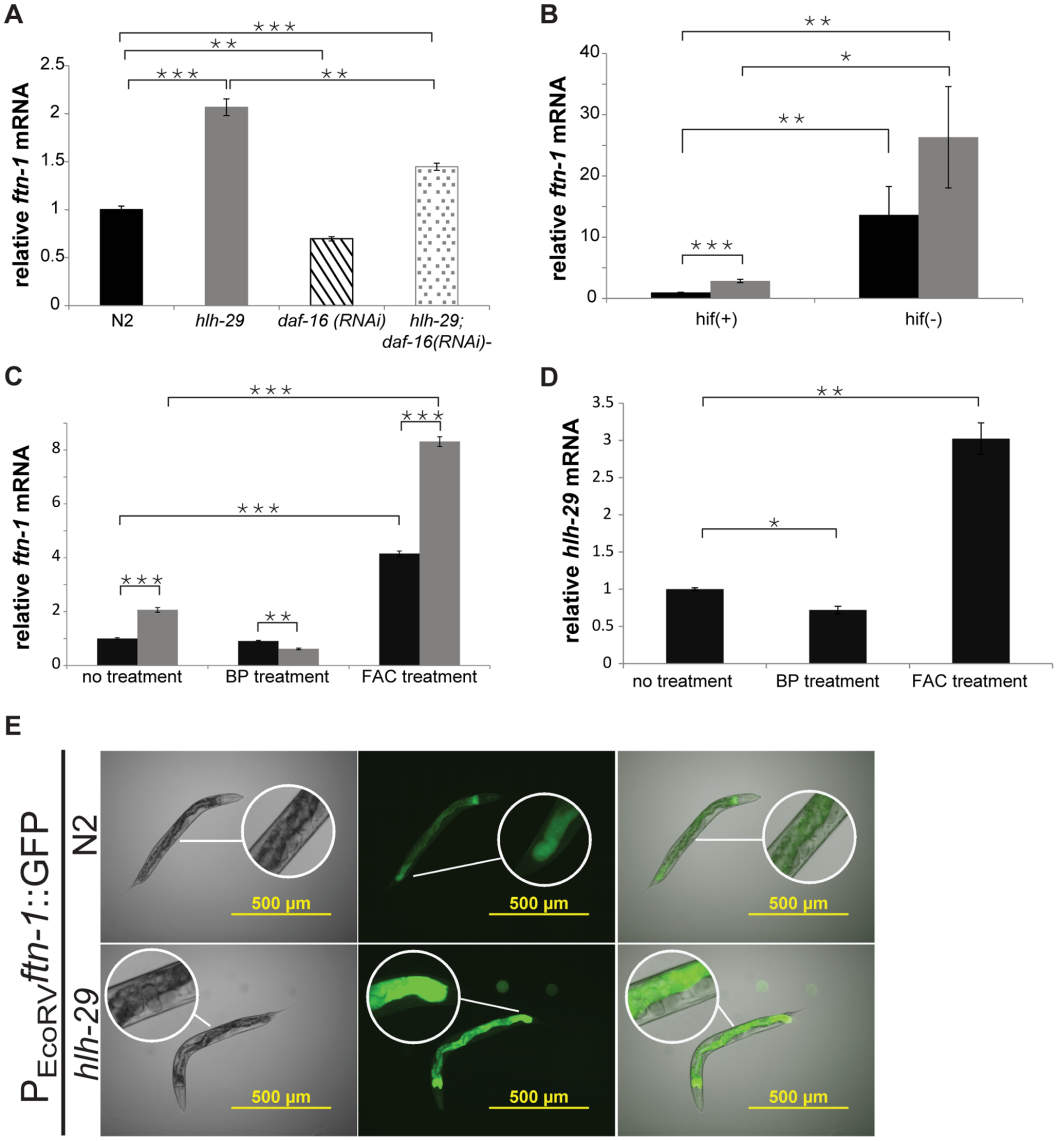
*ftn-1* mRNA Levels in Adult Stage Animals. A) RT-qPCR measurements of *ftn-1* mRNA under normal iron conditions in indicated genetic backgrounds, relative to levels in wild-type animals. B) RT-qPCR measurements of *ftn-1* mRNA levels in wild-type animals and *hlh-29* mutants subjected to *hif-1* RNAi. C) RT-qPCR measurements of *ftn-1* mRNA in wild-type animals and *hlh-29* mutants after treatment with BP or FAC. D) RT-qPCR measurements of *hlh-29* mRNA levels in wild-type animals after treatment with BP or FAC. E) Expression of an integrated transgenic reporter of *ftn-1* activity in young adult wild-type animals and *hlh-29* mutants. In panels A - D, error bars represent standard error of the mean (SEM), wild-type = black bars; *hlh-29* = grey bars; *daf-16* = striped bars; *hlh-29*;*daf-16* = spotted bars. *P-value <0.05, **P-value <0.005, *** -value <0.0005).

We used RT-qPCR to measure *ftn-1* mRNA levels extracted from young adult animals. As shown previously, *ftn-1* mRNA levels significantly decreased in animals with silenced *daf-16* activity [38], and as expected from our microarray analysis, increased significantly in *hlh-29* mutants (Figure 2A). In control reactions (Figure S1A) using the same mRNAs, levels of *acs-2* and *aqp-1*, but not of *aak-1*, were altered in *hlh-29* mutants. DAF-16 and HIF-1 work through parallel pathways to regulate *ftn-1* activity. Using bacterial mediated RNA interference, we found that silencing *daf-16* (Figure 2A) or *hif-1* (Figure 2B) in *hlh-29* mutants could partially suppress or further elevate, respectively, the elevated *ftn-1* mRNA levels. These results suggest that HLH-29 mediates *ftn-1* mRNA levels through a pathway that is distinct from both DAF-16 and HIF-1. We also generated transgenic lines carrying an P*ftn-1*::*gfp* transcriptional reporter. In wild-type animals carrying the integrated transgene, the reporter is variably expressed in the intestine: expression in young larvae was distributed throughout the intestine, but was confined to the dorsal and ventral cells of intestinal rings II and IX in most L4 and adult stage animals. As expected from the mRNA studies, transgene expression in *hlh-29* mutants was stronger and more widely distributed (Figure 2E).

In wild-type animals, *ftn-1* mRNA levels decreased by approximately 20% upon exposure to 20 µM of the metal chelator, 2,2-dipyridyl (BP), and increased by approximately 400% upon supplementation with 24 mM iron (ferric ammonium citrate, FAC) (Figure 2C). We found that, like in wild-type animals, *ftn-1* levels in *hlh-29* mutants decreased and increased upon BP exposure and with FAC supplementation, respectively, indicating that HLH-29 is not involved in the iron-dependent regulation of *ftn-1*. We also found that *ftn-1* mRNA levels were higher in *hlh-29* mutants than in wild-type animals, even with FAC supplementation, though in both cases the fold increase in expression was the same. *hlh-29* mRNA levels also responded to reduced and increased iron levels (Figures 2D), which further suggests that HLH-29 does not regulate the iron responsiveness of *ftn-1*.

**Figure 3:**
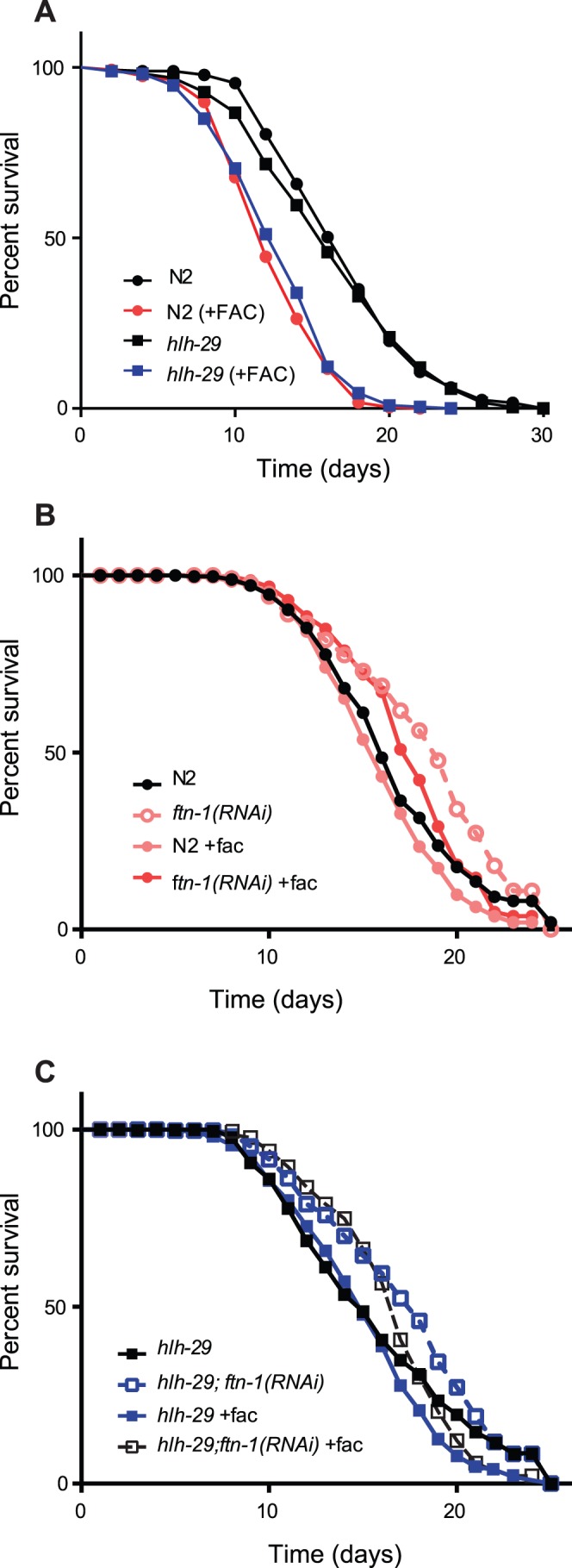
Lifespan Assays. A) The lifespans of wild-type animals and *hlh-29* mutants are similarly affected by 24 mM FAC. (P-value <0.001). B) *ftn-1* RNAi increases the lifespan of wild-type animals under normal growth conditions and in the presence of excess iron. C*) ftn-1* RNAi increases the lifespan of *hlh-29* mutants grown under normal growth conditions and in the presence of excess iron.

Supplementing the *C. elegans* growth medium with FAC effectively increases the amount of free iron in the worms growing on the medium, which can have a detrimental effect on lifespan [59]. We used lifespan assays to determine if the increased *ftn-1* mRNA levels would make *hlh-29* mutants less sensitive than wild-type animals to high iron levels. As expected from previous studies, the lifespan of wild-type animals grown in the presence of 24 mM (6mg/mL) FAC was significantly shorter than the lifespan of animals cultured under normal iron conditions (Figure 3A, Table 2). The lifespan of *hlh-29* mutants was similarly affected by FAC supplementation, despite producing twice the amount of *ftn-1* mRNA under both growth conditions. To determine if increased *ftn-1 *mRNA levels widens the threshold of FAC resistance in *hlh-29* mutants, we measured the lifespan when exposed to 8 mM, 10 mM, 12 mM, or 15 mM FAC. As shown (Figure S2A, Table S5), the lifespan of N2 animals was affected at every concentration tested, with more adverse effects as the FAC concentration increased. This result was different from previous reports that FAC treatments below 15 mM have no significant effect on wild-type lifespan [59]. FAC supplementation also affected the lifespans of *hlh-29* mutants at every concentration tested (Figures S2B, Table S5), and surprisingly, *hlh-29* animals were more sensitive to the FAC concentrations below 24 mM than were wild-type animals. These results suggest that increased *ftn-1* mRNA levels results in increased, rather than decreased, sensitivity of *C. elegans* lifespan to high levels of free iron. To test this possibility, we compared the lifespans of animals grown in the presence of 12 mM FAC and subjected to either *ftn-1* or control RNAi. Reduction of *ftn-1* activity increased the lifespans of both wild-type animals and *hlh-29* mutants when cultured under either normal or high iron conditions (Figure 3B,C and Table S6).

**Table 2:** Lifespan Measurements With or Without 24 mM FAC.

Strain	Concentration of FAC	BiologicalReplicate	Total # 0f Animals toDie/or to be Censored	Median Life Span of All Animals (days)	P-value Log-Rank Test compared to N2 animals
N2	0 mM	1	86/0	18	
		2	80/20	16	
		3	79/17	20	
		**all**	**245/37**	**18**	
	24 mM	1	76/4	12	<0.0001
		2	90/10	12	<0.0001
		3	71/29	12	<0.0001
		**all**	**237/43**	**12**	**<0.0001**
*hlh-29*	0 mM	1	69/2	16	0.0863
		2	81/19	16	0.5189
		3	77/23	18	0.4389
		**all**	**227/44**	**16**	**0.3046**
					
	24 mM	1	72/0	13	<0.0001
		2	81/19	12	0.0002
		3	72/27	14	<0.0001
		**all**	**225/46**	**14**	**<0.0001**

Under low iron conditions, wild-type animals cease to produce ferritin, a process that likely prevents further reduction of labile iron pools in the cell (Figure 2C, [58]). As a result, wild-type animals grown in the presence of BP develop normally, while *hif-1* mutants, who constitutively express *ftn-1*, are developmentally arrested and have reduced viability under low iron conditions [37]. We found that, like wild-type animals, *hlh-29* mutants developed normally when exposed to 20 µM BP (Figure S3A), a result that is consistent with the reduced *ftn-1* mRNA levels in BP-treated *hlh-29* mutants (Figure 2C).

### *hlh-29* Mutants are More Resistant to Peroxide Stress Than Wild-Type Animals

**Figure 4:**
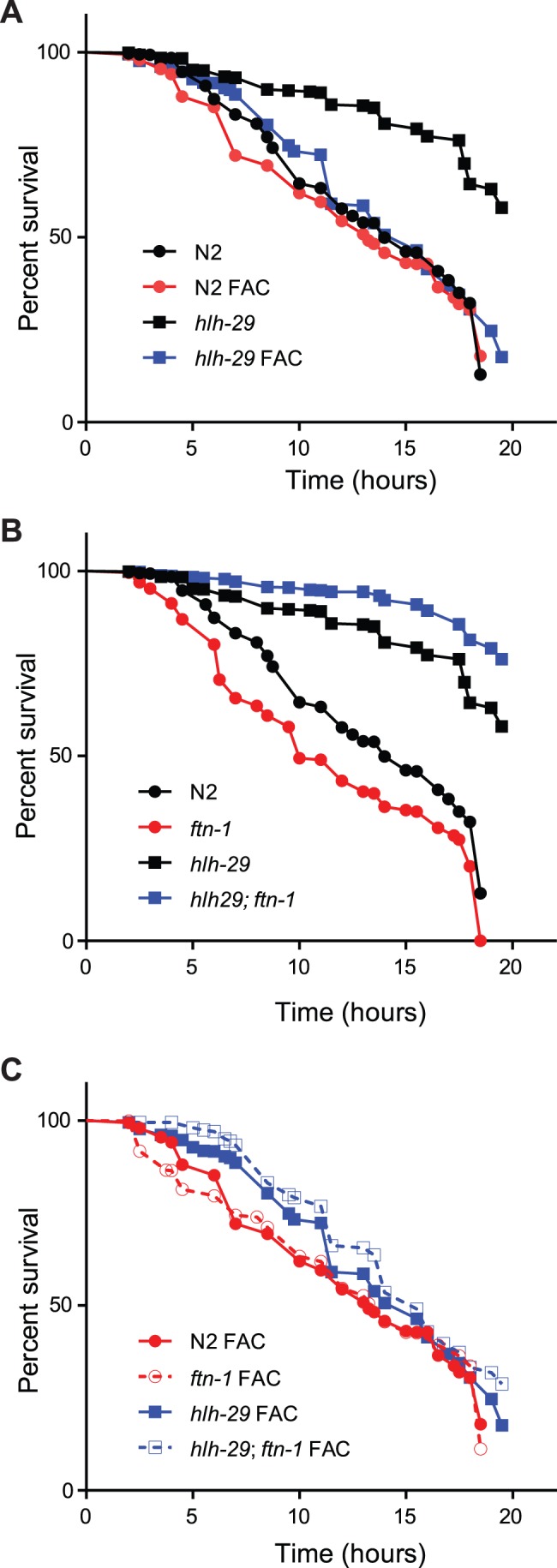
t-BOOH Survival. A) *hlh-29* mutants show increased resistance to 10 mM t-BOOH when compared to wild-type animals under normal growth conditions or when medium is supplemented with FAC. B) The survival of wild-type animals exposed to 10 mM t-BOOH decreases upon treatment with *ftn-1* RNAi, while the survival of *hlh-29* mutants increases. C) *ftn-1* RNAi does not affect t-BOOH survival in the presence of FAC in wild-type animals, but slightly increases survival of *hlh-29* mutants. See supplementary tables and methods for statistical information.

Increased iron levels can cause oxidative stress in *C. elegans* without affecting lifespan, an effect that can be ameliorated by over-producing FTN-1 [59]. Therefore, we expected that the increased *ftn-1* mRNA levels in *hlh-29* mutants would be accompanied by increased resistance to hydrogen peroxide induced oxidative stress. As shown in Figure 4A and Table S7, *hlh-29* mutants survived longer than wild-type animals when exposed to 10 mM tert-butyl hydroperoxide (t-BOOH), as expected. We found, as expected, that subjecting wild-type animals to *ftn-1*(RNAi) resulted in slightly, but significantly decreased resistance to peroxide stress (Figure 4B, Table S7). Loss of *hlh-29* rescued the decreased resistance phenotype, as *hlh-29*;*ftn-1*(RNAi) animals are significantly more resistant to peroxide stress than *ftn-1*(RNAi) animals (Figure 4B).

Interestingly, *hlh-29*;*ftn-1*(RNAi) animals were also more resistant to peroxide stress than *hlh-29* mutants. This result was unexpected but suggested that other genes targeted by HLH-29 are involved in regulating the oxidative stress response under normal iron conditions. Therefore, we wondered if the increased *ftn-1* mRNA in *hlh-29* mutants affected resistance to peroxide stress under high iron conditions. As shown in Figure 4A, both wild-type animals and *hlh-29* mutants showed increased sensitivity to oxidative stress when simultaneously exposed to 24 mM FAC, though the change in wild-type animals was very small. Interestingly, the survival of wild-type animals exposed simultaneously to peroxide stress and excess iron was not affected by *ftn-1* RNAi; however, the survival of *hlh-29*; *ftn-1 (RNAi)* mutants slightly, but significantly increased (Figure 4C, Table S7). Similarly, both wild-type animals and *hlh-29* mutants showed increased sensitivity to oxidative stress when exposed to the iron-chelator deferoxamine (Figure S3B), though the increased sensitivity in *hlh-29* mutants was not significant. Together, these results again demonstrate that HLH-29 dependent regulation of *ftn-1* is not dependent on iron levels.

### HLH-29 Likely Binds to Recognition Sequences Upstream of the *ftn-1* IDE

**Figure 5:**
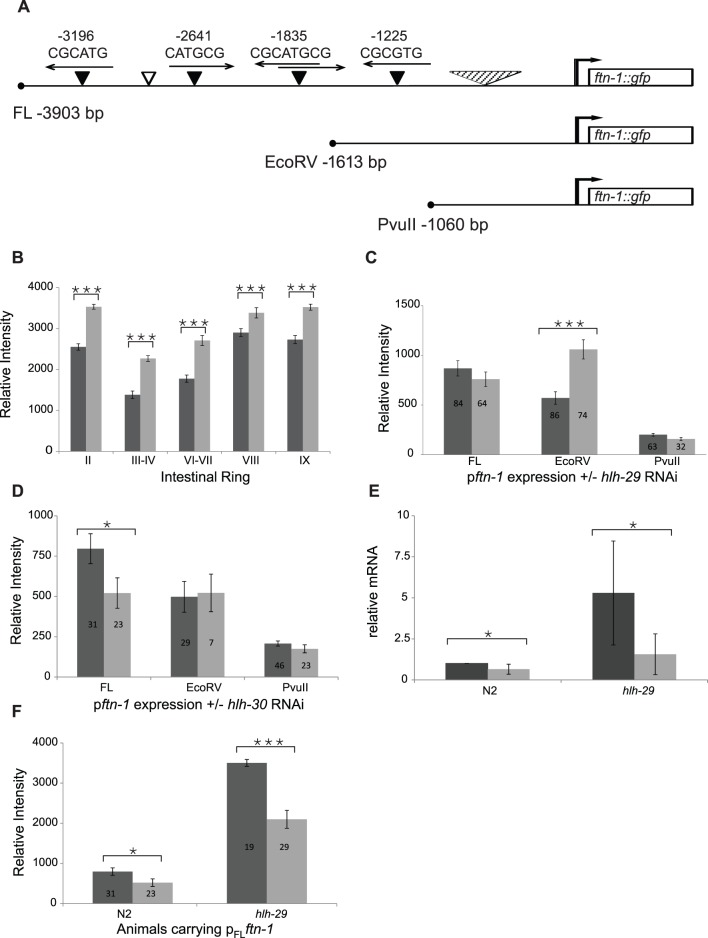
HLH-29 Regulates *ftn-1* Expression. (A) Schematic representation of the *ftn-1* transgenes used in this study. Empty triangle, predicted HLH-30 binding site (CACGTG); filled triangle, predicted HLH-29 binding sites (CATGCG or CACGCG) with arrows indicating the corresponding orientations; striped triangle, predicted IDE region containing binding sites for GATA and HRE types of transcription factors. B) Relative intensity of the expression from integrated P_EcoRV_ *ftn-1::GFP* in the indicated intestinal rings of wild-type animals (dark bars, N = 61) and *hlh-29* mutants (light bars, N = 72). C) Relative intensities of the indicated *ftn-1* reporters in L4 stage, wild type animals subjected to control RNAi (dark bars) or *hlh-29* RNAi (light bars) RNAi. D) Relative intensities of the indicated *ftn-1* reporters in L4 stage, wild type animals subjected to control RNAi (dark bars) or *hlh-30* RNAi (light bars) RNAi. E) RT-qPCR measurements of *ftn-1* mRNA in wild-type animals and *hlh-29* mutant subjected to control RNAi (dark bars) or *hlh-29* RNAi (light bars). F) Relative intensities of the full length *ftn-1* reporter in L4 stage, *hlh-29* mutants subjected to control RNAi (dark bars) or *hlh-30* RNAi (light bars) RNAi. Error bars represent SEM, *P-value <0.05, **P-value <0.005, *** P-value <0.0005).

**Figure 6:**
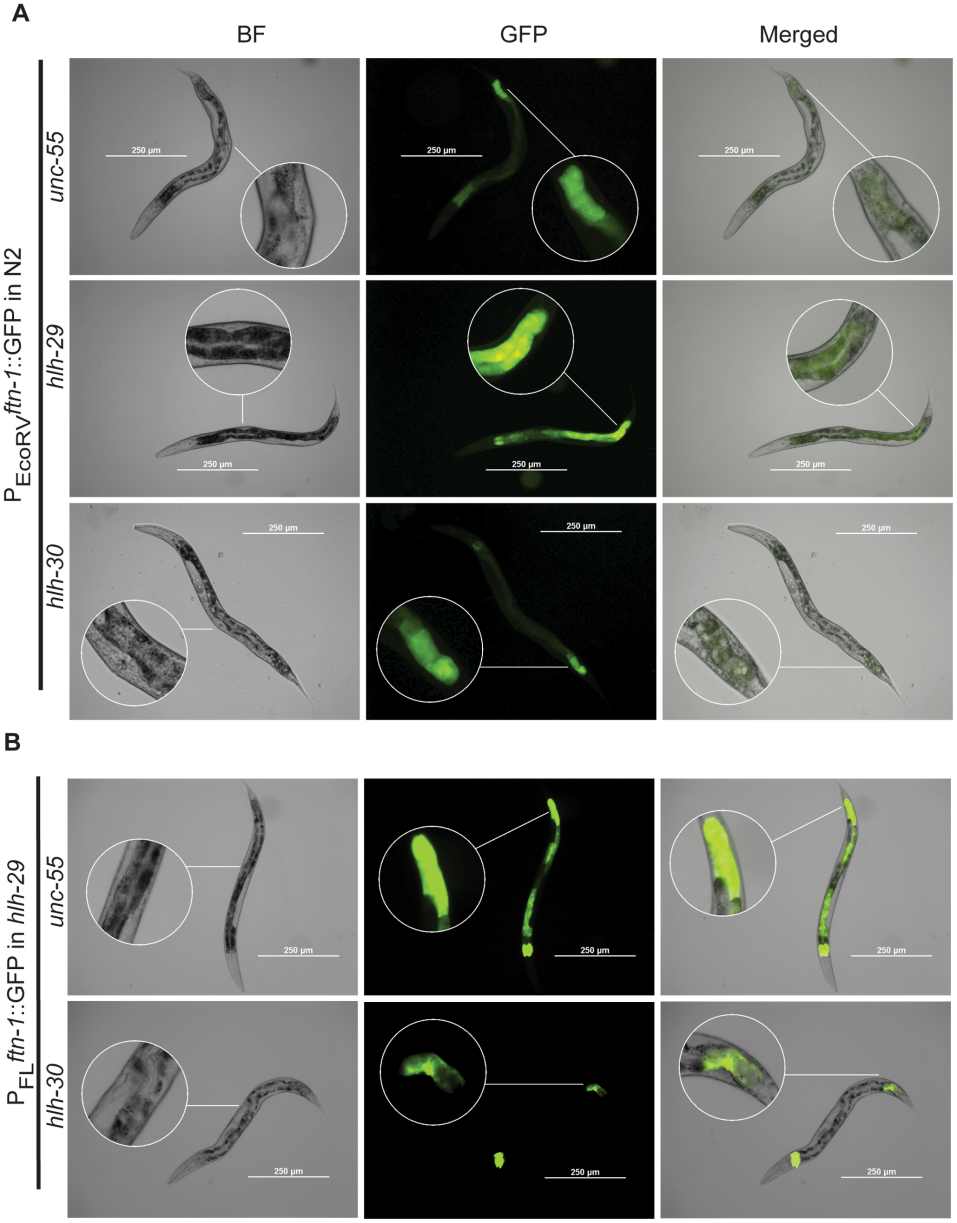
*ftn-1* Expression Profile in Wild Type Nematodes. Stable transgenic lines bearing the EcoRV *ftn-1* promoter covering 1060 bp upstream and 19 bp downstream of the translation start were examined for expression using fluorescence microscopy as described in Methods. Effects of *hlh-29* or *hlh-30* on *ftn-1* promoter activities were achieved by feeding bacterial strains producing dsRNA for either *hlh-29* or *hlh-30* since embryonic stages. Magnified regions emphasize the vulval region in the bright field (BF) images and the posterior intestinal *ftn*-1 expression in the GFP and merged images.

There are four predicted binding sites for the HLH-29 homodimer within the *ftn-1* promoter, three of which are located greater than 1500 base pairs upstream of the translation start (Figure 5A). The integrated transgene shown in figure 2, P_EcoRV_ *ftn-1*::GFP, contains the IDE site and the most promoter proximal HLH-29 binding site. As shown in Figure 5B, expression increased significantly in each of the intestinal rings of transgenic *hlh-29* mutants compared to transgenic wild-type animals. This result is consistent with the mRNA studies, and suggests that HLH-29 recognizes sequences in the *ftn-1* promoter to regulate its activity. To further test this possibility, we generated a full-length reporter, P_fl_ *ftn-1*::GFP, which included all four of the predicted HLH-29 binding sites, and a truncated reporter, P_PvuII_ *ftn-1*::GFP, which eliminated all HLH-29 sites. Expression of these reporters was quantitated in L4 stage wild-type animals subjected to control or *hlh-29* RNAi. We predicted that if HLH-29 binds to the predicted sequences, that removing them would eliminate the effects of *hlh-29* RNAi on reporter activity. The total activity of P_fl_ *ftn-1*::GFP was slightly, but not significantly affected by *hlh-29* RNAi (Figure 5C), possibly because of other regulatory elements present in the distal promoter that affect HLH-29 activity. The expression of P_EcoRV_ *ftn-1*::GFP, increased by approximately 2.75 fold when transgenic animals were subjected to *hlh-29 *RNAi (Figure 5C and 6A), a result that was consistent with the expression of the integrated transgene in wild-type and mutant animals. Importantly, *hlh-29* RNAi did not affect the expression of a reporter that had all four *hlh-29* binding sites removed (P_PvuII_ *ftn-1*::GFP). Taken together, these results suggest that HLH-29 may bind directly to the *ftn-1* promoter.

We found a single HLH-30 recognition sequence within the *ftn-1* promoter, located between two oppositely oriented HLH-29 recognition sequences approximately 2800 bp upstream of the translation initiation site (Figure 5A). The expression of P_FL_ *ftn-1*::GFP, but not of P_EcoRV_ *ftn-1*::GFP or P_PvuII_ *ftn-1*::GFP, was significantly reduced in transgenic wild-type animals subjected to *hlh-30* RNAi (Figure 5D). This result correlates with previous reports that HLH-30 activates *ftn-1* expression [38], and, together with our finding that *ftn-1* mRNA levels are repressed by HLH-29, is consistent with the reciprocal regulation pattern seen for other target genes shared by HLH-30 and HLH-29 (Table S4). We used RT-qPCR to measure *ftn-1* mRNA levels in *hlh-30* (RNAi), *hlh-29*, and *hlh-29*; *hlh-30* (RNAi) mutants. As would be expected from epistatic interactions between two antagonistic genes in a single pathway, *ftn-1* levels increased in *hlh-29* mutants, but decreased in *hlh-30* (RNAi) and *hlh-29*; *hlh-30* (RNAi) mutants (Figure 5E). Similarly, P_FL_ *ftn-1*::GFP expression was lower in *hlh-29*(tm284) mutants subjected to *hlh-30* RNAi than in those subjected to control RNAi (Figure 5F, 6B). Together these results suggest that at least part of the HLH-29 dependent regulation of *ftn-1* under normal growth conditions occurs upstream of HLH-30.

## Discussion

### The HLH-29 Regulatory Network

The REF-1 family member HLH-29 is activated in response to Notch signaling in early *C. elegans* embryos [17], and consistent with this early activation, is predicted to regulate genes needed for development [48]. In larval and adult animals, HLH-29 is expressed in chemosensory neurons, intestines, spermatheca, and vulva muscles, and has been shown to affect fat accumulation, reproduction, and brood size [21], [22]. Here, we have identified genes in the HLH-29 post-embryonic regulatory network. We show that, in young adult animals, the network includes genes involved in regulating iron homeostasis, fatty acid metabolism, and oxidative stress response, many of which are co-expressed with *hlh-29* in the intestine. We also show that many HLH-29 targets are also targets of DAF-16, the insulin/IGF-1 signaling transcription factor that integrates multiple pathways to control lifespan, stress response, and fat storage. Together these results suggest a role for HLH-29 in regulating organismal homeostasis. These results also raise the intriguing possibility that HLH-29 selectively and differentially regulates DAF-16 target genes in response to varying environmental cues. Because HLH-29 has not been reported as a target of DAF-16, and neither was the transcriptional activity of *daf-16* affected in *hlh-29* mutants, we suggest that HLH-29 acts at these genes through a pathway that is distinct from the DAF-16 pathway.

A role for HLH-29 in regulating organismal homeostasis correlates with the inclusion of HLH-30 in the HLH-29 regulatory network. The expression of *hlh-30*, which encodes an ortholog of TFE3, a mammalian transcriptional factor that regulates metabolic genes, is repressed by HLH-29 in young adult animals, as is the expression of 19 other genes that are activated by HLH-30. Interestingly, we found that loss of HLH-30 increases the transcriptional activity of *hlh-29*, suggesting that a negative feedback loop exists between these two transcription factors. In addition to providing genetic evidence of an epistatic relation between the two proteins, our characterization of the *ftn-1* promoter shows that, at some HLH-30 targets, HLH-29 may exert both an indirect and direct influence on gene expression.

### HLH-29, Ferritin Synthesis, and Oxidative Stress

**Figure 7:**
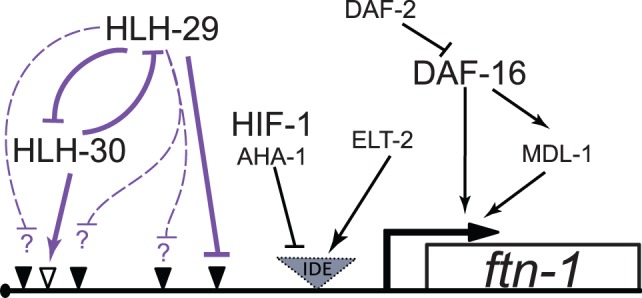
Schematic Representation of the Regulatory Network for *ftn-1* Expression. The previously established regulatory elements are presented with black arrows and the newly established elements from this study are presented with blue arrows. Empty triangle, predicted HLH-30 binding site; filled triangle, predicted HLH-29 binding site; striped triangle, predicted IDE region.

In mammals, ferritin production is regulated in response to iron, as well as in response to oxygen and nitric oxide. Iron-dependent regulation of mammalian ferritin is primarily at the post-transcriptional level via RNA binding proteins that target ferritin mRNAs to proteasomes or to ribosomes [24]; however, oxygen mediated regulation of ferritin synthesis occurs at the transcriptional level [33], [60], [61]. The regulation of ferritin synthesis in *C. elegans* is believed to occur exclusively at the transcriptional level [34], [62], and iron-dependent regulation of *ftn-1* expression is well characterized [35], [37]. The current model involves repression of *ftn-1* activity under low and normal iron conditions by HIF-1 [37] and predicts that transcriptional activation involves competition between HIF-1 and an unidentified bHLH protein for a hypoxia response element (HRE) located within the IDE. There is no information available about oxygen-mediated regulation of ferritin synthesis *in C. elegans*. DAF-16 also transcriptionally regulates *ftn-1* through a pathway separate from HIF-1 [59], which tentatively links ferritin production to insulin signaling. Here we present evidence of direct regulation of *ftn-1* activity by HLH-29, via a pathway that is separate from both HIF-1 and DAF-16 (Figure 7).

Our data raise a number of questions about the biological role of HLH-29 in regulating ferritin synthesis. We found that the increased *ftn-1* mRNA levels of *hlh-29* mutants did not affect lifespan under normal or excess iron conditions; in fact, the increased levels of *ftn-1* mRNA appeared to increase the sensitivity of the lifespans to lower concentrations of exogenous iron. Similarly, our results, including the similar responses of the *ftn-1* and *hlh-29* transcriptional activities to altered iron levels, suggest that HLH-29 is not involved in the iron-dependent regulation of *ftn-1* activity. This is unlike bHLH proteins known to regulate iron homeostasis in other systems. For example, bHLH proteins are required in plants for the negative regulation of genes needed in response to iron deficiency [63], [64], [65] and in tumor cells for the iron-dependent up-regulation of ferritin expression [66], [67].

A number of the genes in the HLH-29 regulatory network are also required for oxidative stress response, and so we wondered if HLH-29 acts to coordinate oxidative stress with ferritin synthesis. Consistent with this possibility, our data show that *hlh-29* mutants are more resistant than wild-type animals to oxidative stress caused by hydrogen peroxide exposure, even in the presence of altered iron levels. We found that silencing *ftn-1* expression in wild-type animals causes them to become less resistant to peroxide stress under normal iron, though their resistance to peroxide stress is unaffected by *ftn-1* silencing under high iron. These results with wild-type animals are consistent with studies in plants [26], [68] and in human cell lines [32], [69], and with previous reports that overexpression of *ftn-1* in otherwise wild-type *C. elegans* increases their resistance to peroxide stress [59]. They are also in line with the model that increased ferritin reduces the availability of labile iron pools, which in turn helps to minimize the generation of reactive oxygen species in the presence of hydrogen peroxide.

Surprisingly, we found that silencing *ftn-1* expression in *hlh-29* mutants significantly increases, rather than decreases, the peroxide stress resistance under both normal and high iron conditions. Though difficult to interpret, our measurements of *ftn-1* mRNA levels in *hlh-29* mutants exposed to peroxide stress, support the possibility that RNAi against *ftn-1* in these animals suppresses a toxic effect of excess ferritin. Recent reports in other systems show, for example, that increased ferritin levels in the presence of hydrogen peroxide [70], [71] or visible light [72] is detrimental to the cell because of increased lipid peroxidation. It is also possible that the increased sensitivity of *hlh-29*;*ftn-1*(RNAi) mutants is the result of HLH-29′s influence on genes required for oxidative stress response. For example, transcriptional regulation of the ferritin gene during oxidative stress in mammals is regulated through a response element that also mediates expression of glutathione-S-transferases, NAD(P)H:quinone reductases, and metallothionine [33]. Similar genes are found in the HLH-29 regulatory network, including the mitochondrial NADP transhydrogenase, NNT-1, and the glutathionine-S-transferases, GST-10, GST-15, and GST-19. Taken together, these results suggest that HLH-29 is a common regulatory denominator in the link between oxidative stress and ferritin synthesis in *C. elegans*.

## Materials and Methods

### Nematode Strains and Maintenance

The strains used were N2 Bristol wild-type [73] and TM284, *hlh-29(tm284)*. Culture growth and maintenance at 20°C and synchronization by alkaline hypochlorite treatment were as previously described [74]. For food source, NGM plates were either seeded with bacterial strain OP50 or with the strain HT115 containing the appropriate vectors for RNAi. For RNAi, worms were synchronized, allowed to hatch on unseeded NGM agar without peptone, and then moved to NGM agar seeded with HT115 producing dsRNA for either the control gene *unc-55* or for the test genes *daf-16*, *hlh-29*, *hlh-30*, and *hif-1* as previously described [75]. For iron supplementation, ferric ammonium citrate [(FAC) – C_6_H_8_O_7_·xF3^+^·yNH_3,_ Fisher Scientific] was dissolved in water, filter sterilized, adjusted to pH 7.0 and added to sterile molten NGM agar to the final concentration specified [35]. For iron chelation, 2,2-dipyridyl [(BP) – C_10_H_8_N_2_, Sigma-Aldrich ] or deferoxamine mesylate salt [(DF) – C_25_H_48_N_6_O_8_ ·CH_4_O_3_S, Sigma-Aldrich] was added to a final concentration of 20 µM or 100 µM, respectively.

### Gene Expression Microarray

Total RNA was extracted from early adult stage populations; animals used for this assay carried between two and five fertilized embryos in the uterus. Gene expression microarray, including probe preparation, hybridization, fluidics run and chip scan, was performed by Georgia State University DNA/Protein Core Facility. Global gene expression in synchronized populations of *tm284* animals was compared to expression in N2 (wild-type) animals using GeneChip *C. elegans* Genome Array (Affymetrix). Data collection was carried out using GCOS 1.4 software (Affymetrix). Data analysis was performed using GeneSpring *GX 11* Software (Agilent, Palo Alto, CA), and probe intensity values were normalized using Robust Multichip Average (RMA)-algorithm. The quality controls on samples and on probe sets were performed stepwise to detect the outlying samples and the poor probe sets. The Principal Components Analysis (PCA) score plot and hybridization controls plot were applied for sample detection. Those probe sets passing minimal detection cutoffs and quality control measurements subsequently were used for statistical analysis. The T-test was performed to find the candidates for differential expression, and genes with significant signal level between different conditions (p<0.05) were collected. In addition, fold change analysis were performed on the genes with significant expression, and all genes showing greater than two-fold change were considered putative targets.

### Functional Analysis

Gene Ontology (GO) analysis was performed using the Database for Annotation, Visualization and Integrated Discovery (DAVID), version 6.7, to cluster related target genes [39], [40]. Additional GO terms and functional information for putative targets were assigned based on Wormbase annotations [76]. Interactions and connectivity between target gene products were identified using the Search Tool for the Retrieval of Interacting Genes/Proteins [41] and redrawn manually.

### Gene Expression Analysis

Total RNA extraction, cDNA synthesis, and real-time PCR were carried out as previously described [77] except total RNA was extracted from late L4/young adult animal populations (48 to 54 hours after feeding from L1 stage at 20°C). mRNAs were checked for genomic DNA contamination using qPCR with the endogenous control primer probe sets in the absence of reverse transcription. cDNA synthesis reactions were performed with 2.0 or 3.0 µg of total RNA in 10µL or 20µL reactions, respectively. Real-time PCR assays were performed with Taqman Gene Expression Assays (Applied Biosystems) for *ftn-1* (Ce02477612_g1), *hlh-29* (Ce02508182_g1), *hlh-30* (Ce02462542_m1), *acs-2* (Ce02486192_g1), *aqp-1* (Ce02421795_g1), *daf-2* (Ce02444343_m1), *aak-1* (Ce02406988_g1), and *daf-16* (Ce02422843_m1) using relative quantitation with normalization against *pmp-3* (Ce02485188_m1) as the endogenous control [78], [79]. For validation of microarray results, total RNA was extracted in triplicate from mixed stage populations, and cDNA synthesis was performed in 50 µL reaction volumes containing either 10.0 µg or 25 µg of total RNA. RT-qPCR assays were performed either with Taqman Gene Expression Assays or by using Sybr® green for detection of amplicon, using relative quantitation with normalization against the endogenous control gene *pmp-3* [78]. Additional primer/probe sequences are available upon request.

### Lifespan Assays

N2 and *hlh-29(tm284)* animals were synchronized to L1 stage and then fed OP50 at 20°C for 48 hours until reaching late L4 stage. Hermaphrodites were visually confirmed to be at L4 stage, then were transferred to seeded NGM plates with or without FAC at the indicated concentrations, and scored for survival either daily (during the egg-laying period) or every two days. Day of transfer was considered to be t = 0, and animals were scored as dead if they failed to respond to at least three touches with either a platinum wire or an eyelash. *hlh*-29(tm284) mutants have a variable exploding vulva phenotype that appeared to be enhanced by FAC supplementation; these animals and those with the “bag of worms” phenotype were censored in these assays. Lifespan survival comparisons were done individually for each of three biological replicates using the log rank test in GraphPad Prism 5 (GraphPad Software, Inc.), and were then repeated with the data from all biological reps combined into one experiment. Combined data are presented in the figures, while individual and combined data are presented in the tables. We found that survival plots of the lifespans of treated and untreated animals show disproportional hazards, and so used both the Mantel-Cox and the Gehan-Breslow-Wilcoxon tests to determine significance [80].

### Peroxide Stress Assays

Synchronized, L4 stage hermaphrodites were placed onto seeded NGM plates containing 10 mM tert-butyl hydrogen peroxide [(t-BOOH) – (CH_3_)_3_COOH, Sigma-Aldrich], with or without 24 mM FAC. Animals were incubated at 22°C and scored for survival at 1.5 to 2 hour intervals. NGM plates containing t-BOOH (+/−FAC) were poured under a chemical hood on the same day as the assay, allowed to solidify with lids open for one hour before being seeded with concentrated, log phase OP50. The bacteria were allowed to absorb into the media for an additional hour before 10 to 15 animals were placed onto each plate. Animals that failed to respond after repeated prodding with a platinum wire were moved to one location, and were then tested again at least 10 minutes later. Animals that failed to respond both times were scored as dead, and those that crawled off the plates or under the agar were censored. Assays were repeated for three separate trials, and were analyzed individually and then collectively using the log rank test in GraphPad Prism.

### Delayed Development Assay

Synchronized L1 stage animals were collected from NGM (-peptone) plates into sterile water, added in one to five µL increments to assay plates containing 20 µM BP and then counted while still swimming in the liquid. Animals were maintained at 20°C and inspected daily until greater than 50% of control treated N2 animals reached or exceeded late L4 stage. At this time, the percentage of animals reaching L4 stage was determined for all animal populations. The experiment was performed in triplicate, and significance was determined using single factor ANOVA. The data presented represent the percentage of treated animals to reach L4 stage relative to the percentage of control treated N2 animals, which was set to 100% regardless of the actual number reaching adulthood (% L4 stage test animals out of total number on plate/%L4 stage control animals out of total on plate).

### Transgenic Line Construction

The full length *ftn-1* promoter covering 3903 bp upstream and 19 bp downstream of the initiator ATG was amplified from *C. elegans* gDNA using the following primers: Pftn-1F 5′- TTT GGA TCC TGT AGG GTT TGA TTG TGG TTT GCT TC-3′ and Pftn1R- TTT TAC CGG TTT GAC GAG CTA GAG ACA TGA CGA TTT AC-3′. The PCR amplified product was subcloned into BamHI and AgeI sites on the pPD95_67 vector (a gift from A. Fire). Truncated forms of the *ftn-1* promoter covering 1613 bp and 1060 bp upstream the coding region were generated by digestion with the restriction enzymes EcoRV and PvuII, respectively. All plasmids were verified by DNA sequencing and then microinjected into N2 and *tm284* animals using an injection mixture consisting of 20 ng/µl *ftn-1* promoter and 100 ng/µl pRF4 (*rol-6*). Only stable transgenic lines with high transmission rates greater than 75% were selected for further studies. The transgene P_EcoRV_ *ftn-1*::GFP was integrated into *hlh-29(tm284)* mutants using UV irradiation as previously described [81], which were subsequently backcrossed 5 times and then crossed to wild-type, N2, animals. Homozygous wild-type animals in the F3 generation were confirmed by PCR genotyping of the *hlh-29* locus.

### Measurement of *ftn-1* Promoter Activity

Stable transgenic lines were examined for expression and fluorescence intensity. Effects *hlh-29* or *hlh-30* on *ftn-1* promoter activities were achieved by feeding transgenic animals that were otherwise wild-type with bacterial strains producing dsRNA for either *hlh-29* or *hlh-30*. Because of difficulties with yeast contamination in our RNAi libraries, animals were cultured on nematode growth medium (NGM) plates supplemented with 1 mM isopropylthio-β-galactoside (IPTG), 12.5 µg/ml tetracycline and 35 µg/ml ampicillin. For imaging, animals were anesthetized with 0.1% levamisole and mounted on 1% agarose pads. Images were captured using a 10× objective with a Nikon Eclipse 80i microscope equipped with a Nikon Coolsnap CCD camera, with a 1.00 s exposure time for all conditions. Fluorescence intensity was quantitated adapted from previously reported methods [82]. Anterior, central, and posterior intestinal regions correspondingly roughly to intII, intIII-IV, intVI-VII, intVIII, and intIX, respectively, were marked as regions of interest (ROI) for intensity measurements using the software NIS-Elements, version 3.2 (Nikon). All measurements were recorded using the raw data image without any processing/adjustment to allow comparison of expression levels in different animals. Expression is reported as the average adjusted GFP intensity, which is the average GFP intensity of three intestinal regions minus the average background intensity per animal.

## Supporting Information

Figure S1 **mRNA levels in adult stage animals.** Encapsulated Postscript. A) Control RT-qPCR measurements of indicated gene transcripts in wild-type (black bars) and *hlh-29(tm284)* (grey bars) animals. Expression of daf-2, daf-16, and aak-1 was not significantly affected when measured by microarray or by RT-qPCR. B) *hlh-29* mRNA levels increase slightly in wild-type animals subjected to hlh-30 RNAi (P-value <0.01), but not in *hlh-29(tm284)* mutants.
(EPS)

Figure S2 **Lifespan assays in wild-type animals and** ***hlh-29*** **mutants subjected to increasing FAC concentrations.** Encapsulated Postscript. A) Lifespans comparisons for wild-type animals grown in the absence of FAC (black circles) or in the presence of 8 mM, 10 mM, 12 mM, or 15 mM FAC. B) Lifespans comparisons for *hlh-29* mutants grown in the absence of FAC (black squares) or in the presence of 8 mM, 10 mM, 12 mM, or 15 mM FAC.
(EPS)

Figure S3 ***hlh-29*** **mutants are not affect by iron chelators.** Encapsulated Postscript. A) *hlh-29* mutants develop at the same rate as wild-type animals when exposed to 20 µM BP. Percent Δ represents the change in the number of animals to reach L4 stage when grown in the presence versus in the absence of BP, see methods for more details. B) Treatment with100 µM DF increases the sensitivity of wild-type animals to 10 mM t-BOOH (p-value 0.0004), but not of *hlh-29* mutants (p-value 0.4314).
(EPS)

Table S1 Genes changed by 2.0 fold or greater in *hlh-29* mutants. Excel spreadsheet.
(XLSX)

Table S2 Gene Ontology (GO) of genes affected in *hlh-29* mutants. Excel spreadsheet.
(XLSX)

Table S3 HLH-29 targets regulated by DAF-16, Oxidative Stress, or Aging. Excel spreadsheet.
(XLSX)

Table S4 HLH-29 targets regulated by HLH-30. Excel spreadsheet.
(XLSX).

Table S5 Lifespan measurements with increasing FAC. Microsoft Word Document.
(DOC)

Table S6 Influence of *ftn-1* RNAi and FAC on lifespan. Microsoft Word Document.
(DOC)

Table S7 Influence of *ftn-1* RNAi and FAC supplementation on peroxide toxicity. Excel spreadsheet.
(XLSX)
