# Supplementary material for: Genome-Wide Microarrray Analysis Reveals Roles for the REF-1 Family Member HLH-29 in Ferritin Synthesis and Peroxide Stress Response
Source: PLoS One. 2013 Mar 22;8(3):e59719. doi: 10.1371/journal.pone.0059719 (PMC3606163; doi:10.1371/journal.pone.0059719)
Supplement: Table S5 — Lifespan measurements with increasing FAC. Microsoft Word Document. (DOC) [file pone.0059719.s008.doc]

**Table S5: Lifespan measurements with increasing concentrations of FAC**

1. Lifespan in Wild-Type Animals

|  |  |  |  | **P-value vs N2**  **0 mM** | |  | **P-value vs *hlh-29***  **at same [FAC]** | |
| --- | --- | --- | --- | --- | --- | --- | --- | --- |
| **FAC** | **REP** | **Deaths/**  **Censored** | **Mean LS** | **Mantel Cox** | **Wilcoxon** |  | **Mantel Cox** | **Wilcoxon** |
| 0 mM | 1 | 96/4 | 18 |  |  |  | 0.0473 | 0.0546 |
|  | 2 | 86/11 | 13 |  |  |  | 0.4405 | 0.6081 |
|  | 3 | 78/22 | 13 |  |  |  | 0.8478 | 0.2744 |
|  | **all** | **260/37** | **15** |  |  |  | **0.0642** | **0.2187** |
|  |  |  |  |  |  |  |  |  |
| 8 mM | 1 | 95/7 | 16 | 0.0002 | <0.0001 |  | 0.0188 | 0.0027 |
|  | 2 | 91/9 | 15 | 0.0071 | 0.1129 |  | 0.0007 | 0.0004 |
|  | 3 | 93/7 | 13 | 0.3835 | 0.2481 |  | 0.4939 | 0.0799 |
|  | **all** | **279/23** | **15** | **0.0003** | **0.1005** |  | **0.0006** | **<0.0001** |
|  |  |  |  |  |  |  |  |  |
| 10 mM | 1 | 93/7 | 16 | 0.0002 | 0.0003 |  | 0.001 | 0.0031 |
|  | 2 | 92/8 | 15 | 0.1782 | 0.5756 |  | 0.0021 | 0.0012 |
|  | 3 | 88/12 | 13 | 0.6088 | 0.1767 |  | 0.5887 | 0.0953 |
|  | **all** | **273/27** | **15** | **<0.0001** | **0.0666** |  | **0.1450** | **0.4087** |
|  |  |  |  |  |  |  |  |  |
| 12 mM | 1 | 93/4 | 16 | <0.0001 | <0.0001 |  | 0.0019 | 0.0051 |
|  | 2 | 78/22 | 13 | 0.0102 | 0.278 |  | <0.0001 | <0.0001 |
|  | **all** | **171/26** | **16** | **<0.0001** | **0.0174** |  | **<0.0001** | **<0.0001** |
|  |  |  |  |  |  |  |  |  |
| 15 mM | 2 | 92/8 | 13 | 0.0259 | 0.8142 |  | 0.619 | 0.3327 |
|  | 3 | 94/6 | 13 | 0.8048 | 0.2317 |  | 0.4335 | 0.0992 |
|  | **all** | **279/18** | **14** | **<0.0001** | **0.002** |  | **0.1144** | **0.0079** |
|  |  |  |  |  |  |  |  |  |

1. **Lifespan in *hlh-29* Mutants**

|  |  |  |  | **P-value vs *hlh-29***  **0 mM** | |
| --- | --- | --- | --- | --- | --- |
| **FAC** | **REP** | **deaths/**  **censored** | **mean LS** | **Mantel Cox** | **Wilcoxon** |
| 0 mM | 1 | 91/9 | 18 |  |  |
|  | 2 | 74/26 | 13 |  |  |
|  | 3 | 88/11 | 15 |  |  |
|  | **all** | **253/46** | **15** |  |  |
|  |  |  |  |  |  |
| 8 mM | 1 | 86/15 | 14 | <0.0001 | <0.0001 |
|  | 2 | 79/22 | 13 | 0.0419 | 0.1626 |
|  | 3 | 84/18 | 13 | 0.0384 | 0.0214 |
|  | **all** | **249/55** | **13** | **<0.0001** | **<0.0001** |
|  |  |  |  |  |  |
| 10 mM | 1 | 85/15 | 18 | 0.074 | 0.0916 |
|  | 2 | 69/31 | 13 | 0.0122 | 0.2745 |
|  | 3 | 86/14 | 13 | 0.0399 | 0.0362 |
|  | **all** | **240/60** | **14** | **0.8905** | **0.4824** |
|  |  |  |  |  |  |
| 12 mM | 1 | 91/9 | 14 | <0.0001 | <0.0001 |
|  | 2 | 73/27 | 11 | <0.0001 | 0.0013 |
|  | **all** | **155/45** | **13** | **<0.0001** | **<0.0001** |
|  |  |  |  |  |  |
| 15 mM | 2 | 78/23 | 13 | 0.1569 | 0.3798 |
|  | 3 | 81/20 | 13 | 0.2508 | 0.078 |
|  | **all** | **250/48** | **14** | **<0.0001** | **<0.0001** |
|  |  |  |  |  |  |
