# Supplementary material for: Genome-Wide Microarrray Analysis Reveals Roles for the REF-1 Family Member HLH-29 in Ferritin Synthesis and Peroxide Stress Response
Source: PLoS One. 2013 Mar 22;8(3):e59719. doi: 10.1371/journal.pone.0059719 (PMC3606163; doi:10.1371/journal.pone.0059719)
Supplement: Table S6 — Influence of ftn-1 RNAi and FAC on lifespan. Microsoft Word Document. (DOC) [file pone.0059719.s009.doc]

**Table S6: Influence of *ftn-1* RNAi and FAC on Lifespan**

|  |  |  |  | **P-value vs Strain (X) when treated with *ftn-1*(RNAi)**** | |
| --- | --- | --- | --- | --- | --- |
| **Strain (Condition)** | **REP** | **Deaths Censored** | **Mean Survival** | **Mantel**  **Cox** | **Wilcoxon** |
| N2 | 1 | 89/10 | 15 | < 0.0001 | <0.0001 |
|  | 2 | 76/24 | 17 | 0.0668 | 0.4198 |
|  | **all** | **165/34** | **17** | **0.0002** | **0.0003** |
|  |  |  |  |  |  |
| N2 (+FAC) | 1 | 97/6 | 15 | 0.0005 | <0.0001 |
|  | 2 | 86/14 | 17 | 0.8082 | 0.2662 |
|  | **all** | **183/20** | **15** | **0.0098** | **0.0002** |
|  |  |  |  |  |  |
| *ftn-1*(RNAi) | 1 | 93/9 | 19 |  |  |
|  | 2 | 71/29 | 19 |  |  |
|  | **all** | **164/38** | **19** |  |  |
|  |  |  |  |  |  |
| *ftn-1*(RNAi) (+FAC) | 1 | 86/14 | 17 |  |  |
|  | 2 | 84/16 | 17 |  |  |
|  | **all** | **170/30** | **17** |  |  |
|  |  |  |  |  |  |
| *hlh-29* | 1 | 86/13 | 15 | 0.0142 | 0.0005 |
|  | 2 | 90/11 | 15 | 0.0539 | 0.0756 |
|  | **all** | **176/24** | **15** | **0.0021** | **0.0002** |
|  |  |  |  |  |  |
| *hlh-29* (+ FAC) | 1 | 90/10 | 15 | < 0.0001 | <0.0001 |
|  | 2 | 92/8 | 17 | 0.0123 | 0.1579 |
|  | **all** | **182/18** | **17** | **0.3342** | **0.0173** |
|  |  |  |  |  |  |
| hlh-29;*ftn-1*(RNAi) | 1 | 85/15 | 17 |  |  |
|  | 2 | 88/12 | 19 |  |  |
|  | **all** | 173/27 | **19** |  |  |
|  |  |  |  |  |  |
| *hlh-29;ftn-1*(RNAi)  (+ FAC) | 1 | 94/7 | 17 |  |  |
|  | 2 | 95/5 | 17 |  |  |
|  | **all** | **189/12** | **17** |  |  |
|  |  |  |  |  |  |
| ****X = strain indicated to the left of the table** | | |  |  |  |
